# A Survey of Intestinal Parasites of Domestic Dogs in Central Queensland

**DOI:** 10.3390/tropicalmed2040060

**Published:** 2017-11-21

**Authors:** Simone Gillespie, Richard S. Bradbury

**Affiliations:** School of Health, Medical and Applied Sciences, Central Queensland University, North Rockhampton, QSD 4702, Australia; simone.gillespie17@hotmail.com

**Keywords:** dogs, canine, domestic, parasites, zoonoses, hookworm, Queensland, Australia

## Abstract

Australia has a very high rate of dog ownership, which in some circumstances may lead to exposure to zoonotic parasitic diseases from those companion animals. Domestic dog faecal samples (*n* = 300) were collected from public spaces and private property in the greater Rockhampton (Central Queensland) region and tested for intestinal helminths and protozoa by direct microscopy, two flotation methods and a modified acid-fast stain for cryptosporidia. Intestinal parasites detected included hookworms (25%), *Cystoisospora ohioensis* complex (9%), *Blastocystis hominis* (3%), *Giardia duodenalis* (3%), *Spirometra erinacei* (1%) and *Toxocara canis* (1%), *Sarcocystis* spp. (2%), *Cryptosporidium* spp. (2%) and *Cystoisospora canis* (1%). One infection each with *Trichuris vulpis*, *Dipylidium caninum* and a protozoa belonging to the *Entamoeba histolytica* complex were identified. Sheather’s sucrose centrifugal flotation was more sensitive than saturated salt passive flotation, but no single test detected all cases of parasitic infection identified. The test methodologies employed are poor at recovering larva of *Strongyloides stercoralis*, *Aleurostrongylus abstrussis* and eggs of cestodes such as *Echinococcus granulosis*, so the potential presence of these parasites in Central Queensland domestic dogs cannot be excluded by this survey alone.

## 1. Introduction

Australia has one of the highest rates of pet ownership in the world, with 63% of households owning a pet. Dogs remain the most common companion animal, accounting for 39% of all domestic pets. It is estimated that there are 4.2 million pet dogs in Australia, correlating to 19 dogs for every 100 people [1]. The level of pet care in Australia is high, with strict legislations in place to ensure responsible pet ownership. One of the largest factors in pet ownership is parasitic infection control, which represents a major source of income for veterinarians around the country [1,2]. The close relationship between humans and their pet dogs can lead to transmission of intestinal parasitic diseases between these two species, with several parasites of dogs causing zoonotic infection in humans. Several broad surveys of domestic dog parasites have been conducted in Australia, but only one since the turn of the century. This national survey did not publish the data collected by state or climatic region [2]. No survey of all intestinal parasites of domestic dogs in an urban tropical Australia has been published until now.

Examples of zoonotic parasites reported in Australian domestic dogs that may lead to patent infection in humans following direct transmission include *Strongyloides stercoralis*, *Ancylostoma ceylanicum*, *Trichuris vulpis*, *Giardia duodenalis* and *Blastocystis hominis* [3,4,5,6,7,8,9,10,11]. Other parasites of domestic dogs may not reach patency in humans, but immature or larval stages have the capacity to cause significant disease in human hosts. The common dog hookworm, *Ancylostoma caninum*, may cause eosinophilic enteritis or cutaneous larva migrans in humans [3]. Visceral or ocular larva migrans disease caused by *Toxocara canis* may occur via ingestion of embryonated eggs from the environment [3]. 

Currently, there is limited information about the prevalence of canine gastrointestinal parasitic infections in regional and rural areas of Australia, particularly in the tropical North. The purpose of this study was to determine the prevalence and species of intestinal parasites in canines in Central Queensland, a regional and rural area within of the Tropic of Capricorn. The survey was conducted over a twelve-month period throughout the Greater Rockhampton region of Central Queensland (including Rockhampton city, Yeppoon, Cawarral, Gracemere and Emu Park areas), with a population living in both urban and rural residences. A total of 300 canine faecal samples were collected and analysed by microscopy for the presence of intestinal parasites.

## 2. Materials and Methods

### 2.1. Specimen Collection and Faecal Consistency Scoring

Specimen collection occurred once per week between January and October 2015, thereby including the end of the 2014/2015 wet season and the whole of the 2015 dry season. Fresh (based upon appearance and consistency) domestic dog (*Canis lupus familiaris*) faecal samples were collected and analysed from public spaces including parks, beaches and popular walking paths between 5 am and 9 am (*n* = 174). Residential area collections (*n* = 120) were performed with full consent and supervision from the property owner. Six samples were provided incidentally by a veterinary surgery at a time when the researchers were attending to collect ticks taken from animals for a separate study. These were all from domestic dogs held overnight for observation at the surgery. Prior to processing methods, the consistency of each sample was scored using the Purina Faecal Scoring System (Purina Faecal Scoring System for Dogs and Cats, Nestle-Purina Pet Food Co., St. Louis, MO, USA) and note of any mucous, blood, adult helminths or proglottids in the specimen was made. 

### 2.2. Sample Processing and Direct Saline Preparation Microscopy

Gross faecal specimens were used to prepare a direct smear (12.5 mm × 25 mm in size) directly onto a glass microscope slide for later Kinyoun modified acid-fast staining. Samples were mixed with saline to form a slurry, then filtered through cotton gauze to remove larger particulate matter. A direct wet preparation was performed from this saline slurry, observing for the presence of parasitic elements and any motility of same. A portion of the slurry was then centrifuged in two 15 mL conical tubes. The centrifuged faecal deposit was corrected by removal of excess using wooden applicator sticks until the final volume of 1 mL was achieved (equivalent to 1 gram of faeces). Sheather’s sucrose and salt flotation methods then performed upon sample in each of the respective conical centrifuge tubes. 

### 2.3. Preparation of Sheather’s Sucrose and Saturated Salt Solutions

Two flotation solutions were prepared in distilled water as described by Zajac et al. [12]. Briefly, Sheather’s sucrose was prepared as 0.97 mol/L of sucrose (IGA, Canning Vale, Australia) in distilled water; the saturated salt solution was 6.0 mol/L NaCl (IGA, Canning Vale, Australia) in distilled water. The solutions were heated with a magnetic stirrer until completely dissolved. Mixtures were allowed to cool. 6 mL of 37% formaldehyde solution was added as a preservative to the Sheather’s sucrose solution only.

The specific gravity of each batch was controlled using on a laboratory balance. An empty 15 mL plastic centrifuge tube was filled with exactly 2.0 mL of distilled water and weight checked as being 2.0 g. The same tube was then completely emptied and refilled with exactly 2.0 mL of Sheather’s sucrose solution or saturated salt solution. The weights of these solutions were required to be within 2.54 ± 0.02 for Sheather’s sucrose (S.G. 1.27) and 2.40 ± 0.02 for the saturated salt solution (S.G. 1.2) before use. Solutions were stored at room temperature until use.

### 2.4. Saturated Salt Passive Flotation Method

This method was performed as described by Zajac et al. [12]. Approximately 8 mL of saturated salt solution was added to 1 g of centrifuged faecal deposit in a 15 mL conical tube. The tube was then tightly capped and mixed vigorously to homogenise. The tube was then uncapped and placed upright in a test tube rack and topped up with the salt flotation solution to completely fill the test tube, ensuring that a slightly convex meniscus was formed. A 15 × 15 mm coverslip was placed over this meniscus, and the solution was allowed to stand for 15 min. Following this, the coverslip was carefully removed, ensuring that the drop of solution from the meniscus remained attached to the bottom of the coverslip. 

### 2.5. Sheather’s Sucrose Centrifugal Flotation Method

This method was performed as described by Zajac et al. [12], but with top-up of solution following centrifugation, despite a swing rotor centrifuge being employed. Approximately 8 mL of Sheather’s sucrose solution was added to 1 g of centrifuged faecal deposit in a 15 mL conical tube. The tube was then tightly capped and mixed vigorously to homogenise. Tubes were then centrifuged at 500 g for two minutes. After centrifugation, the tubes were then uncapped and placed upright in a test tube rack, followed by topping up with Sheather’s sucrose solution to completely fill the test tube, ensuring that a slightly convex meniscus was formed. A 15 mm × 15 mm coverslip was placed over this meniscus, and the solution was allowed to stand for 12 min. Following this, the coverslip was carefully removed, ensuring that the drop of solution from the meniscus remained attached to the bottom of the coverslip. 

### 2.6. Kinyoun’s Modified Acid-Fast Stain

For the staining and detection of *Cryptosporidium* oocysts, air-dried faecal smears were fixed in absolute methanol for 60 s. Slides were then flooded with Kinyoun’s carbol fuchsin (Sigma-Aldrich, Darmstadt, Germany) for five minutes. Slides were briefly rinsed in 70% ethanol and then washed in tap water before de-staining with 1% acid alcohol (1 mL H_2_SO_4_, 99 mL ethanol) for two minutes. Slides were further washed in tap water before counterstaining with malachite green (Sigma-Aldrich, Darmstadt, Germany; 0.5 g in 100 mL distilled water) for 30 s, followed by washing in tap water and air drying.

### 2.7. Parasite Identification by Microscopy

The entire coverslip of the direct saline and each of the flotation preparations was placed on a microscope slide, and the entire slide was scanned at 100× and 400× magnification. The entire smears of Kinyoun’s modified-acid fast-stained slides were examined at 1000× magnification, with *Cryptosporidium* oocysts identified by colour, size and distinctive morphology in the stain. Parasites were identified by size and characteristic morphology. For differentiation of the *Cystoisospora* species, *Cytoisospora canis* (size > 33 µm) was differentiated from species belonging to the *C. ohioensis* complex (size < 30 µm), based on the size of oocysts [13]. Each coverslip and Kinyoun-stained smear was examined separately by two trained morphologists (SG and RSB). 

### 2.8. Statistical Analysis

Sample size was calculated based upon the hypergeometric distribution equation for sample size, based upon the assumption that the samples collected were random, and that no host was sampled more than once [(1 − (1 − *c*)^1/d^)(*N* − (*d*/2)) + 1] as described by Cannon and Roe [14], where *N* is the population size, *d* is the number of infected hosts in the population, and *c* is the desired confidence level. The relative sensitivity and 95% confidence interval of each method employed was determined, using the combined results of each method as a reference standard. All statistical analyses were carried out in Microsoft Excel.

### 2.9. Ethical Approval

Ethical approval for this study was granted by the Animal Ethics Committee of Central Queensland University (approval number A15/01-325).

## 3. Results

### 3.1. Faecal Survey Results

A total of 300 canine faecal samples were collected between February and November 2015. This sample size allowed determination with 95% confidence that the prevalence of any intestinal parasite not found in our sample is at <1%, and 99% confidence that the prevalence of any parasite not recovered was <1% of the total population [14] of domestic dogs in the Greater Rockhampton region. Of these samples, 120 (40%) were positive for one or more gastrointestinal parasites; in 88 (29%) dogs, a single parasite species was identified; 27 (9%) had dual infection; and five (2%) were infected with three parasite species. Hookworm eggs were the most common parasitic element seen, being found in 24% of faecal samples analysed. Eggs of other helminths were observed only rarely; the *T. canis* or *T. vulpis* eggs detected were not larvated. The *C. ohioensis* complex was the most common protozoan parasite of domestic dogs, found in 9% of samples, while *G. duodenalis* and *B. hominis* were found in 3% of samples. Other protozoan parasites were at individual prevalences below 3% (Table 1).

### 3.2. Faecal Sample Consistency and Appearance

The majority (79%) of faecal specimens collected were of a healthy consistency (score 2–4); only 8% showed signs of diarrhoea (scores 5–6). No specimens collected were from dogs with watery diarrhoea (score 7). One faecal sample contained macroscopically visible mucous strands; this formed, but moist (score = 3) stool contained eggs of hookworms and cyst-like stages of *B. hominis*. Another single faecal samples had visible macroscopically visible blood; this loose and wet sample (score = 5) was found to harbour eggs of hookworm and cysts of *G. duodenalis*. 

### 3.3. Spurious Parasites

Several samples contained mite eggs. Two samples contained eggs resembling those of *Ascaridia* spp., an ascarid intestinal helminth of birds. One sample contained eggs morphologically indistinguishable from those of *Syphacia muris*, an oxyurid intestinal helminth of rodents. 

### 3.4. Comparison of Test Methodologies

The relative sensitivities and 95% confidence intervals for each test for hookworm were: direct saline preparation, 24% (15–35%); passive salt flotation, 83% (72–90%); and Sheather’s sucrose centrifugal flotation, 91% (82–96%). For other parasite species, numbers did not reach a level at which a statistically valid test sensitivity could be achieved. No single method was ideal for the detection of *C. ohioensis* complex oocysts; in many cases, these were detected by one method and not by the other two, without any method being notably superior. There was a notable degree of variation in the sensitivity of each method employed for the detection of parasites. This was noticeable in the protozoa test results, particularly those for *C. ohioensis* complex and *B. hominis,* which were often detected in only one method, but no method seemed markedly superior in detection than another (Table 2). In four cases, *G. duodenalis* cysts were detected in the background malachite green stain of the Kinyoun *Cryptosporidium* stain and not by any other method.

## 4. Discussion

There is a high prevalence of hookworm infection in domestic dogs in coastal Central Queensland. Compared to studies performed in southern temperate regions of Australia, which found hookworm prevalence ranging between 1.9% and 6.7% [2], the Greater Rockhampton region hookworm prevalence is unusually high. However, this reported prevalence is compatible with that found in comparable tropical and warm temperate regions, both in Australia and overseas [2,15,16,17]. Indeed, compared to previous studies of hookworm prevalence in Queensland, such as the 76.3% prevalence in domestic dogs found in 1973 [16] and more recent studies finding up to 100% prevalence in wild and domestic dogs in remote communities [5,18], the prevalence reported here is relatively moderate. The species of hookworms infecting dogs has an important impact on their risk and potential severity as zoonotic pathogens of humans. It is known that *A. caninum*, *A. braziliense* and *A. ceylanicum* may infect dogs in Queensland [18,19]. *A. ceylanicum* is of particular concern due to its demonstrated capacity to cause patent infections in humans in Australia [20]. *A. caninum* may also cause significant disease, with many cases of eosinophilic enteritis caused by this organism having previously been reported in Queensland [21]. Cutaneous larva migrans is a documented zoonoses occurring in Central Queensland [22], acquired by exposure of unprotected skin to the filariform larvae of *A. braziliense* and possibly also *A. caninum.* Without further molecular analysis to determine species, the degree and type of zoonotic disease risks presented by the high hookworm prevalence in domestic dogs in Rockhampton is unclear.

There was a low prevalence of *T. canis* and *T. vulpis* infection. The fact that the eggs seen were morulated, rather than embryonated, demonstrates that these were true infections, rather than egg passage following coprophagy. A risk of visceral or ocular migrans exists from *T. canis* and rarely, infections with *T. vulpis* in humans have been documented [7,8,9]. Given the low prevalence of these two helminths in Central Queensland dogs, the risk of such human infections is low in this region. However, to further minimise risk, it is advisable that specific hygiene practices are followed by dog owners. Attention to the removal and safe disposal of domestic dog faeces, to avoid contamination of the immediate environment with viable eggs, hand washing to reduce the risk of faecal-oral exposure to eggs and regular deworming of domestic dogs, are all suggested as activities reducing the risk of *Toxocara* larva migrans [3], and will be effective in reducing the risk of zoonotic infections from domestic dogs more generally.

Although *G. duodenalis* was the most prevalent intestinal parasite of dogs in some studies in southern, temperate regions of Australia [2], its prevalence in the dogs we tested was only 3%. This also represents a lower percentage prevalence than those previously reported Australian studies, which have been between 7.7% and 30.7% [2]. *G. duodenalis* may infect both humans and dogs; this species has several genetic assemblages (A–G) that are quite host specific [23]. Only two assemblages, A and B, have been found to infect humans in Australia [20,24]. Studies of the genetic assemblages of *Giardia* in Australian dogs have found that almost all were assemblages C or D, with the single assemblage A infection, considered to be a possible anthroponosis [24]. Therefore, the presence of *G. duodenalis* cysts and trophozoites in the faeces of companion animal dogs presents little or no zoonotic risk to the owners or others. 

*Cryptosporidium* oocysts were found in the faeces of several dogs in this study. Based on the findings of a previous Australian study, it may reasonably be assumed that the majority of these are *Cryptosporidium canis* [24]. Although occasional reports do exist of transmission of *C. canis* from dogs to humans [25], given the high level of exposure to this agent from domestic dogs, such cases are quite rare and present only a very limited zoonotic risk. As an example, a study of 2414 human cases of cryptosporidiosis in the United Kingdom found only 0.04% attributable to *C. canis* [26]. 

One *E. histolytica* complex cyst was observed in the faeces of a dog in this study. *E. histolytica* infection has previously been reported from both dogs and humans [27] and may cause significant intestinal disease in both species. The *E. histolytica* complex includes five morphologically identical species of amoeba, of which only *E. histolytica* sensu stricto causes dysentery. Without PCR analysis, the exact species recovered here cannot be determined. 

Cysts of *C. ohioensis* and *C. canis* were recovered from a number of dogs. Although there is no documented zoonotic risk from these coccidia, ingestion of the oocysts may lead to the formation of persistent monozoic tissue cysts in paratenic hosts. Canine *Cystoisospora* are capable of forming such cysts in human cell culture lines [28]. However, this does not necessarily reflect a capacity to form persistent cysts in the tissues of immunocompetent humans, as host immunity plays an important role in parasite biology. Regardless, the potential for such cysts to form in humans, especially in those who are severely immunocompromised or immunosuppressed, cannot be entirely excluded.

A number of specimens examined contained spurious parasites. The finding of *Ascaridia*-like and *S. muris*-like eggs in dog faeces at first seems exceptional, given that dogs are not hosts for these animals. However, such findings are not unusual, representing passage of eggs consumed with the definitive host by the dogs concerned. These dogs are simply passing eggs without any infection being established following meals of definitive host birds (chickens, turkeys, pigeons, some parrots) for the *Ascaridia* or rodents (rats and mice) for *S. muris*. These findings highlight the potential confounding effect of coprophagy on faecal examinations among dogs [29]. Similarly, mite eggs were seen in a number of the samples tested in this study. These are often seen in parasite screens of dog faeces. These eggs closely resemble those of hookworm, but may be differentiated by observing size, as most mite eggs are larger than those of hookworm (>65 µm) and also by examining the embryo, in which developing mite legs may be observed, rather than the development of a hookworm larva.

Methodological limitations were present in this study. The absence of watery samples (Purina Faecal Scoring System score = 7) may be attributed to the fact that the samples used in this study were collected from parks and yards, where such watery samples would seep into the ground and be unobtainable. This may have biased our results away from parasites that may cause watery diarrhoea, such as the *Cryptosporidium* spp. The detection of *G. duodenalis* is best performed by the use of permanently stained slides; not having employed such slides may have reduced the number of *Giardia* infections identified. The absence of larva of *S. stercoralis* and *Aleurostrongylus abstrussis* is attributable to both the flotation methodology employed and the low faecal larval output in both infections. The minimal amounts of faeces examined in the initial wet-preparation microscopy is insufficient to detect infections with these agents and the larvae do not float in saturated salt solution or Sheather’s sucrose and thus would not have been seen in the microscopy. Overall parasite detection, for both helminths and protozoa, was best using the Sheather’s sucrose centrifugal flotation method. Previous studies have determined slightly improved recovery with centrifugal flotation compared to passive flotation in zinc sulphate and Sheather’s sucrose solutions [30,31], although neither method detected all infections when compared to necropsy [31]. Further comparison of each flotation solution used in this study by both passive and centrifugal flotation is warranted. Therefore, the absence of these in the findings of this study should not be interpreted as their absence from the Greater Rockhampton region. Other helminth infections, specifically trematode and cestode, that produce eggs of a higher specific gravity than the flotation solutions will not have been detected by this study. Our detection of cestode infections is likely a gross underestimate, based on the methodology employed. Previous work showing that Sheather’s sucrose centrifugal flotation methods only detected 6.3% of *D. caninum* infections detected on necropsy [31]. Recovery of *Taenia* sp. was similarly poor at 57.3% by centrifugal flotation and 14.3% by passive flotation [31]. This is particularly important in the case of our non-detection of *Echinococcus granulosis*, an extremely pathogenic zoonotic parasite from dogs. The eggs of this species have a specific gravity too high (S.G. = 1.3−1.4) to easily float in the solutions used, and the sensitivity of the methods employed here range between only 3% and 33% for detection of this parasite [32]. Therefore, the results of this study should under no circumstances be interpreted as suggesting that there is no risk for hydatid disease from domestic dogs in Central Queensland. Sampling of dog scats in parks and other public places may have led to a bias towards collections from adult dogs, as smaller puppies are often not walked by owners for the first few months of life. Parasitic infections more prevalent in puppies might have been detected at higher prevalence, had a deliberate stratified age sampling approach been employed.

## 5. Conclusions

Hookworm infection of domestic dogs in the greater Rockhampton region of Central Queensland is common, but not inconsistent with previous studies in tropical urban environments. A limited zoonotic risk exists from domestic dogs in this region, but this cannot be fully determined without further molecular identification of the species recovered. Of the methods employed in this study, Sheather’s sucrose centrifugal flotation is the overall most effective method for the recovery of canine intestinal parasites, but no single method detected all infections. The use of flotation and direct saline preparation methodologies means that accurate estimates of the prevalence of *S. stercoralis, A. abstrussis*, as well as many trematodes and cestodes, including *E. granulosis*, in Central Queensland cannot be inferred based on the results of this study alone.

## Figures and Tables

**Table 1:** Faecal consistency and associated intestinal parasites detected in a survey of domestic dog faecal samples collected in the greater Rockhampton region of Central Queensland between February and November 2015.

	Purina Faecal Consistency Score *														
	1		2		3		4		5		6				
	Hard and Dry		Formed but Soft		Formed but Moist		Formed and Wet		Semi-Formed and Wet		Loose and Wet		Overall Total		
	*n*	(%)	*n*	(%)	*n*	(%)	*n*	(%)	*n*	(%)	*n*	(%)	*n*	(%)	95% C.I.
Faecal samples	49	(16)	126	(42)	86	(29)	23	(8)	13	(4)	3	(1)	300	(100)	
**Helminths**													**85**	**(28)**	**(23–33)**
Hookworms	9	(19)	29	(23)	31	(36)	3	(13)	3	(2)	0		75	(25)	(20–30)
*Spirometra erinacei*	0		0		4	(5)	0		0		0		4	(1)	(0–3)
*Toxocara canis*	0		0		3	(3)	0		0		1	(33)	4	(1)	(0–3)
*Trichuris vulpis*	0		0		0		0		0		1	(33)	1	<1	(0–1)
*Dipylidium caninum*	0		0		1	(1)	0		0		0		1	<1	(0–1)
**Protozoa**													**62**	**(21)**	**(16–26)**
*Cystoisospora ohioensis* ^§^	2	(4)	13	(10)	7	(8)	5	(22)	1	(8)	0		28	(9)	(6–13)
*Blastocystis hominis*	2	(4)	3	(2)	2	(2)	1	(4)	2	(15)	0		10	(3)	(1–5)
*Giardia duodenalis*	0		5	(4)	2	(2)	0		1	(8)	1	(33)	9	(3)	(1–5)
*Sarcocystis* spp.	0		5	(4)	1	(1)	0		0		0		6	(2)	(0–4)
*Cryptosporidium* spp.	0		2	(2)	1	(1)	1	(4)	1	(8)	0		5	(2)	(0–3)
*Cystoisospora canis*	1	(2)	1	(1)	1	(1)	0		0		0		3	(1)	(0–2)
*Entamoeba histolytica* ^§^	0		1	(1)	0		0		0		0		1	<1	(0–2)
**Parasites not seen**	**38**	**(78)**	**74**	**(59)**	**50**	**(58)**	**12**	**(52)**	**4**	**(31)**	**2**	**(67)**	**180**	**(60)**	**(54–66)**

* Purina Faecal Scoring System for Dogs and Cats, Nestle-Purina Pet Food Co, St Louis, Mo. ^§^ These agents each represent a morphologically identical complex of species, *sensu stricto* species cannot be confirmed by morphology alone. C.I.: Confidence interval.

**Table 2:** Comparison of intestinal parasite detection by three test methodologies used in a survey of 300 domestic dog faecal samples collected in the greater Rockhampton region of Central Queensland between February and November 2015.

Results	Parasite Detected by Test Methodology						Comparison of Parasite Detection by Each Test Methodology			
	Saline Prep *n* (% of Total *)		Salt Float *n* (% of Total *)		Sheather’s *n* (% of Total *)		Detected in Saline, but Not Salt Float	Detected in Saline, but Not Sheather’s	Detected in Salt Float, but Not Sheather’s	Detected in Sheather’s, but Not Salt Float
Parasites Detected	46	(31)	93	(63)	105	(71)	22	16	24	39
**Helminths**	22	(26)	70	(82)	73	(86)	5	1	10	15
Hookworms	18	(24)	62	(83)	68	(91)	4	0	7	15
*Spirometra erinacei*	3	(75)	4	(100)	3	(75)	0	0	1	0
*Toxocara canis*	1	(25)	2	(50)	1	(25)	1	1	1	0
*Trichuris vulpis*	0	(0)	1	(100)	1	(100)	0	0	0	0
*Dipylidium caninum*	0	(0)	1	(100)	0	(0)	0	0	1	0
**Protozoa**	24	(39)	23	(37)	32	(52)	17	15	14	24
*Cytoisospora ohioensis* ^§^	15	(54)	7	(25)	17	(61)	13	11	7	17
*Blastocystis hominis*	2	(20)	5	(50)	6	(60)	0	2	3	4
*Giardia duodenalis* ^†^	3	(33)	5	(56)	3	(33)	0	0	2	0
*Sarcocystis* spp.	0	(0)	6	(100)	4	(67)	0	0	2	2
*Cytoisospora canis*	3	(100)	0	(0)	1	(20)	3	2	0	0
*Entamoeba histolytica* ^§^	1	(100)	0	(0)	1	(100)	1	0	0	1
**No parasites identified**	**254**	**(141)**	**212**	**(118)**	**195**	**(108)**	**54**	**62**	**30**	**14**

* Total determined by a combined reference standard of all methods employed. ^§^ These agents each represent a morphologically identical complex of species, *Sensu stricto* species cannot be confirmed by morphology alone. ^†^ Four *Giardia duodenalis* were detected in background stain of Kinyoun acid fast-stained smears only. Saline: direct saline preparation method; Salt float: saturated salt passive flotation method; Sheather’s; Sheather’s sucrose centrifugal flotation method.

## Dedication

This work is dedicated to the memory of Emeritus Professor Rick Speare. As both a Vet and a Medical Doctor, Rick was always involved in parasitology, zoonotic disease and ‘One Health’ research such as the work presented here. We hope that he would feel this paper to be suitable contribution to this edition of *Tropical Medicine and Infectious Diseases* in honour of the great and generous man that Rick was. He is sorely missed in his passing.
